# Chlorate-induced molecular floral transition revealed by transcriptomes

**DOI:** 10.1515/biol-2022-0612

**Published:** 2023-07-29

**Authors:** Songgang Li, Houbin Chen, Jiwang Hong, Xiuxu Ye, Jiabao Wang, Yeyuan Chen, Lei Zhang, Zuanxian Su, Ziqin Yang

**Affiliations:** Tropical Crops Genetic Resources Institute, Chinese Academy of Tropical Agricultural Sciences, Haikou 571101, Hainan, China; College of Horticulture, South China Agricultural University, Guangzhou 510642, Guangdong, China

**Keywords:** *Dimocarpus longan*, transcriptome, KClO_3_, flowering

## Abstract

Flowering in off-season longan (*Dimocarpus longan* L.) can be induced effectively by the application of potassium chlorate (KClO_3_), but the mechanism of the physiological induction is largely unknown to decipher its mechanism and identify genes potentially regulating the process, and comparative analysis via RNA-Seq was performed between vegetative and KClO_3_-induced floral buds. A total of 18,649 differentially expressed genes (DEGs) were identified between control and treated samples. Gene ontology and Kyoto Encyclopedia of Genes and Genomes (KEGG) pathway analysis revealed that DEGs related to plant hormone signal transduction, mitogen-activated protein kinase (MAPK) signaling pathway, starch and sucrose metabolism, and phenylpropanoid biosynthesis were enriched in our data. A total of 29 flowering-related DEGs were identified in our study, such as *APETALA1* (*AP1*), *APETALA2* (*AP2*), *AUXIN RESPONSE FACTOR 3/ETTIN* (*ARF3*), *SQUAMOSA PROMOTER BINDING PROTEIN-LIKE 8* (*SPL8*), *AGAMOUS* (*AG*), and others. The upregulation of *AP2* and *SPL* genes indicates that the age-related pathway is activated and influences the floral induction in KClO_3_-induced longan floral buds by coordinated regulation of genes related to *AP1*, *AG*, and *ARF3*. This study provides a valuable resource for studying molecular mechanisms underlying chlorate-induced floral transition in off-season longan, which may benefit the development and production of off-season tropical/subtropical fruit trees.

## 1 Introduction

Flowering is an important event in plants and can be controlled by environmental factors as well as endogenous factors. Flower induction requires the transition from the vegetative to the reproductive development stage, which is affected by light intensity and quality, photoperiod, low temperature (vernalization), as well as hormones and sugar status in plant cells. A perennial fruit tree longan (*Dimocarpus longan*) belongs to the family Sapindaceae and close relative of lychee (*Litchi chinensis*), which is commonly cultivated in subtropical and tropical countries including Southeast Asia and Australia [1]. Longan fruit is usually consumed for its nutritional and medicinal values [2]. Flower blossoming in longan mostly occurs once during the spring and requires low temperatures and dry conditions for flower bud differentiation. The mature centralized listing of longan has brought challenges to the development of the longan industry. The off-season flowering can be artificially induced by the application of chemicals such as potassium chlorate (KClO_3_) during noninductive temperature conditions [3]. However, the flowering response varies depending on the cultivars and regions and responds to a particular transient development stage of the terminal bud [4].

Due to the extended generation time, the detailed information related to molecular mechanisms of floral induction in longan is limited. The genetic basis of flower induction pathways and meristem development is similar across plant species. A significant amount of studies on model plants and perennial plants (e.g., *A. thaliana*) have identified key pathways and genes associated with floral transition including photoperiod, vernalization, gibberellic acid (GA), aging, etc. These pathways are regulated by floral integron genes such as flowering locus T (*FT*), flowering locus C (*FLC*), *constans* (*CO*), *LEAFY* (*LFY*), and *suppressor of overexpression of constans* (*SOC1*). The FT translocates from leaves to the apical meristem and interacts with another transcription factor encoded by the floral meristem (FM) identity gene *APETALA1* (*AP1*) which targets downstream signals of flower development. The minichromosome maintenance1, agamous, deficiens and serum response factor (MADS)-domain protein, FLC, is a key repressor protein involved in vernalization, which interacts with another transcriptional repressor short vegetative phase (SVP) in *Arabidopsis* under a cold environment. The SVP protein further interacts with positive regulators of the FM development, SOC1, and AGL24. GA is a major player in a range of biological processes (BPs) and regulates the flower development. However, GA has been shown to act as a repressor of flowering in some plants. Two FM identity genes, *LFY* and *AP1*, act in a positive feedback loop and activate floral homeotic genes, which specify the identity of floral organs (sepals, petals, stamens, and carpels). In a proposed floral quartet model, floral homeotic proteins form organ-specific complexes and require another protein SEPALLATA (SEP; SEP1, SEP2, SEP3, and SEP4) for interaction. Moreover, several transcription factors involved in the flower development have been identified, such as MADS-domain transcription factors (TFs), AP2/ERF, MYB, NAC, WRKY, and DREB [5]. It has been reported that longan *FT1* and *FT2* genes have been ectopically expressed in *Arabidopsis* and showed early and late flowering in overexpressing lines (*DlFT1* and *DlFT2*), respectively [6]. Further, *DlAP1* and *DlAP2* overexpressing *Arabidopsis* lines showed varying flowering time phenotypes. Studies showed that flower induction requires the presence of mature leaves, indicating the importance of flowering genes that express in mature leaves such as *FT* [7]. Interestingly, an analytical study on KClO_3_-treated longan revealed that a higher level of GA in shoot tips contributed to flower induction [8]. Many studies to date have been carried out on flowering induction and floral bud development in longan; however, the knowledge related to the molecular mechanism and its regulation during KClO_3_-induced flowering in longan are still evasive [1,6].

In this study, a comparative transcriptome analysis was performed using KClO_3_-treated and untreated off-season longan cultivars at a specific development stage. We ask if the treatment can induce expressions of genes like AP and ARF3 in off-season longan buds, as those in other plant species. We hypothesize that, as physiological induction of flowering has been observed in longan, genes related to flower initiation should be induced with the treatment in longan buds. This is the first comprehensive study to identify the differentially expressed genes and regulatory pathways involved in floral bud induction in longan in response to KClO_3_ application using the RNA-Seq approach. Our results may provide resourceful information related to flower induction and development, which may further be used to improve unstable flowering in subtropical fruit trees.

## 2 Materials and methods

### 2.1 Plant material and treatments

**Figure 1:**
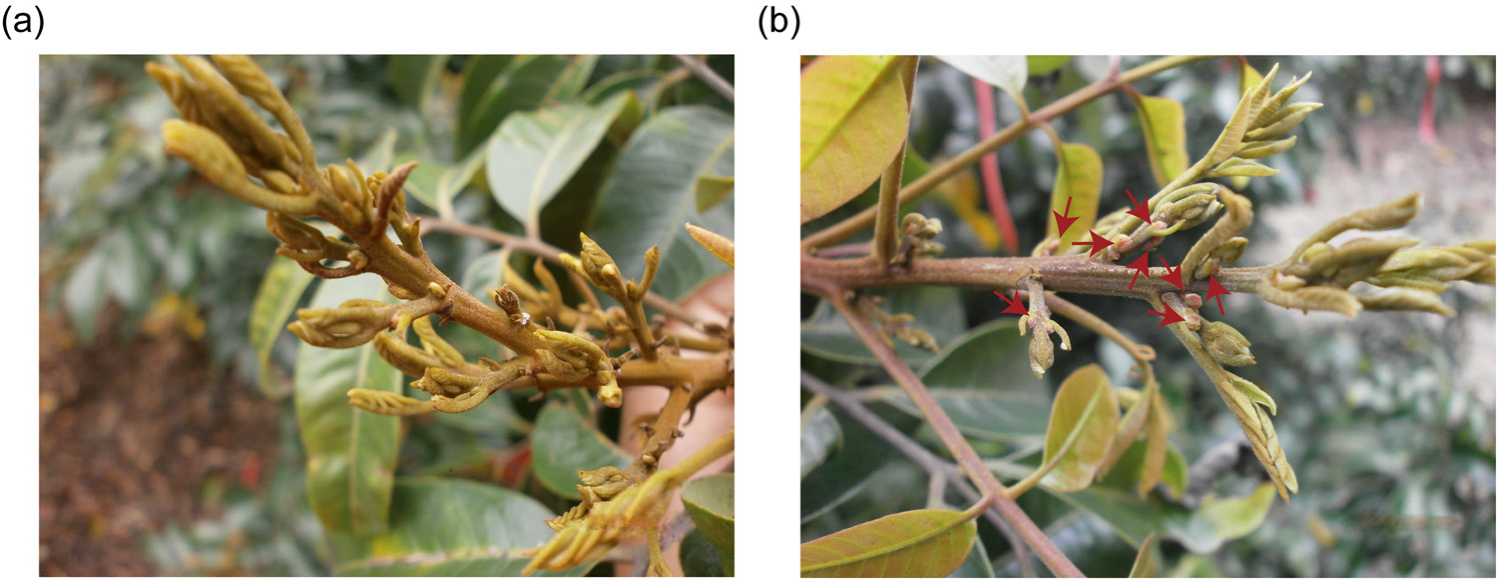
Shoots and flowers of longan without (a) and with (b) treatment of potassium chlorate (KClO_3_). Arrows indicate the emergence of floral buds after KClO_3_ application.

The experiments were conducted on the longan trees grown in a noninductive environment to avoid flower induction due to vernalization. At the national cultivar improvement center, 10- to 11-year-old tropical fruit trees, “Chuliang” trees, of similar size and developmental stage were selected for artificial flower induction treatment. Longan trees were treated with KClO_3_ (300 g per tree) as a solid drench. The generated flower buds were collected after 4 weeks of the treatment. Trees without treatment were set as a control (Figure 1). All samples were collected in triplicate. The collected samples were immediately frozen in liquid nitrogen and stored at −80°C.

Total RNA was isolated using a plant RNA extraction kit according to the manufacturer’s instructions and treated with RNase-free DNase I to remove any DNA contamination. The quantity and quality checks for RNA samples were performed on Nanodrop ND1000 spectrophotometer and Agilent 2100 Bioanalyzer (Agilent Technologies, USA). A total of six RNA samples (three biological replicates for each treatment) with RNA integrity number value >8 were used for complementary DNA (cDNA) library preparation and sequencing.

### 2.2 Library construction and RNA sequencing

In total, six cDNA libraries were generated and sequenced, three biological replicates of 0 weeks and three replicates of 4 weeks after the treatment, using the Illumina HiSeq 2500 PE150 platform (Illumina Inc. CA, USA). The Illumina-generated paired-end raw reads were preprocessed with FastQC (v.0.11.3). The adapter sequences and low-quality reads were filtered by AdaperRemoval-v2 (version 2.2.0) followed by rRNA removal. The Q20, Q30, and GC content of the clean reads were determined. The high-quality clean reads were aligned to the longan genome and gene model downloaded from the website (http://gigadb.org/dataset/100276).

### 2.3 Differential gene expression analysis

The Kallisto program (v0.46.2) was used to count the number of reads mapped and to estimate the gene expression. The normalized gene expression level was computed as transcript per kilobase (kb) per million reads mapped (TPM). Differential expression analysis between samples was analyzed using the DESeq2 R package (v1.30.1). Genes with log2 fold change ≥1 or <−1 with adjusted *p*-value ≤0.05 were used as cutoff criteria for differentially expressed genes (DEGs). The *p*-values were adjusted using Benjamini and Hochberg’s false discovery rate method.

### 2.4 Functional annotation of DEGs

The functional annotation of longan genes was performed with the diamond (v2.0.8.146) tool by aligning the protein sequences to the NCBI nonredundant (Nr) database (*e-*value = 1 × 10^−3^). Gene ontology (GO) analysis was performed using InterproScan (v5.51–85.0). The GO and KEGG enrichment analysis for differentially expressed genes (DEGs) was carried out using ClusterProfiler (v3.18.1) R package. GO terms with an adjusted *p*-value ≤0.05 were depicted as significantly enriched. The ggplot2 (v3.3.3) was used to draw the volcano plot (Figure 3a) and MA plot (Figure 3b), and GO enrichment graph. The correlation coefficient for the correlation matrix was calculated using the corrplot (v0.88) R package.

### 2.5 Quantitative real-time polymerase chain reaction

Total RNAs were extracted from buds with the treatment of KClO_3_ (300 g per tree) after 0, 1, and 4 weeks, respectively, using the RNAprep Pure Plant Kit (TIANGEN, Beijing, China), following the manufacturer’s instructions. cDNA was synthesized using Reverse Transcriptase M-MLV kit (Takara, Beijing, China) according to the manufacturer’s instructions. Quantitative reverse transcription polymerase chain reaction (qRT-PCR) was performed using a TianLong 988 Real-Time PCR System (Tianlong Technologies, China) according to the manufacturer’s instructions. A total of 6 l of DNase/RNase-free water, 11 μl of Green Real-Time PCR master mix, 2 μl of diluted cDNA product, and 1 μl of gene-specific primers were added to each reaction mixture. Three biological replicates were used for each tissue and three technical repeats for each biological replicate. The thermal cycle was set as follows: denaturing at 95°C for 30 s, denaturing at 95°C for 15 s, and annealing and elongating at 58°C for 30 s with 45 cycles. The following are the primers used. AP1: CTTTGTGATGCTGAGGTTGCT (forward) and GGATTTTGCTGCTCCCATTGT (reverse); ARF3: GCATTTAGGGGCAGTCAAGAT (forward) and GGCATAGAAGTGGCTTACATTGG (reverse); BOP1: TAGCCAAACACCTGCCCATC (forward) and GCCTTCACCACTTCTCTGCT (reverse). The Actin7 gene (CCAGCCATCTCTCATCGGAA (forward) and GTCGGCAATACCAGGGAACA (reverse) are primers) was used as an internal reference for the normalization of gene expression. The relative expression levels were calculated using the 2^−ΔΔCt^ method.

### 2.6 Data availability

All the raw sequencing data have been deposited in the NCBI Sequence Read Archive (SRA) database under the accession number PRJNA731294.

## 3 Results

### 3.1 RNA sequencing and mapping

**Figure 2:**
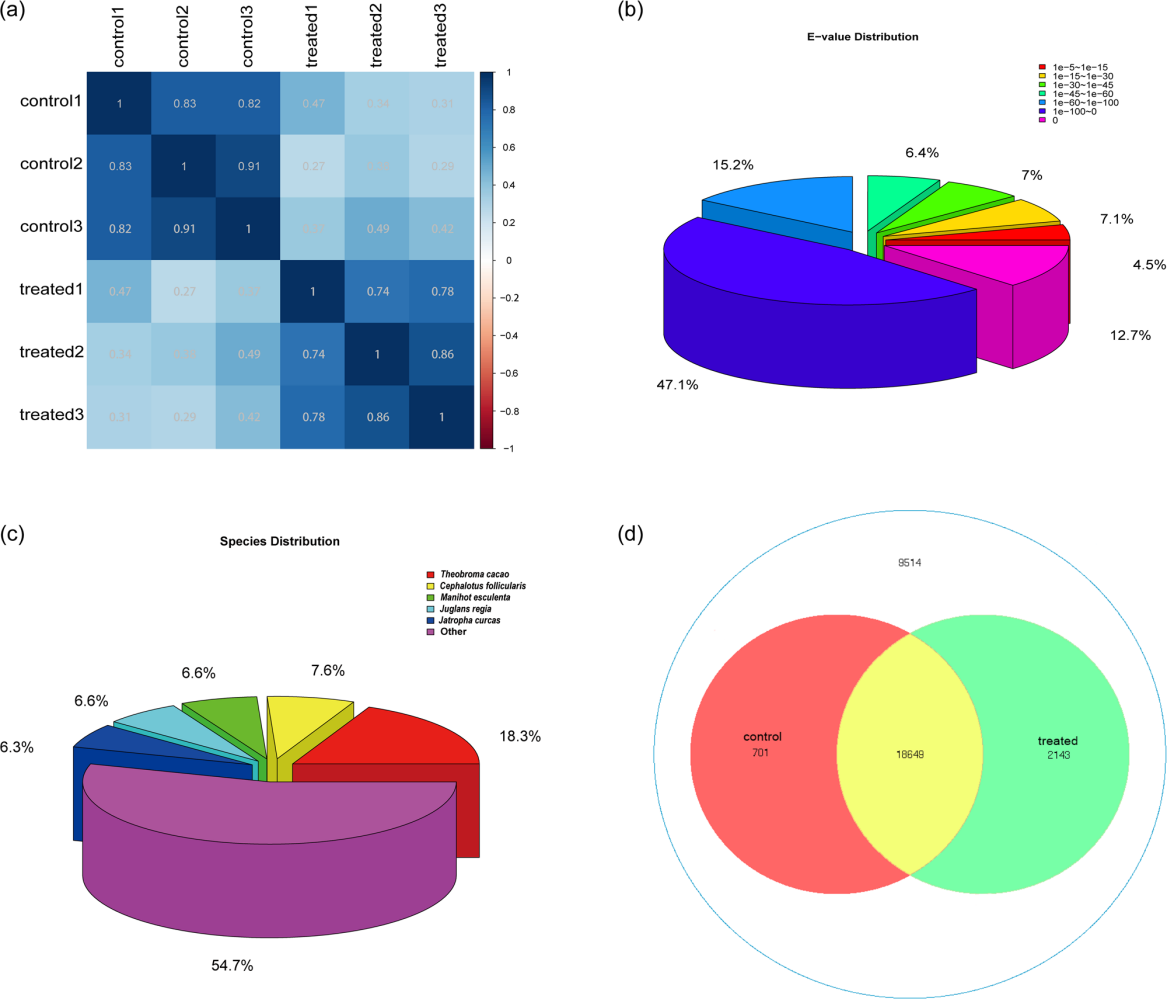
Summary of RNA-Seq results. (a) Heatmap showing correlation of expression between samples, suggesting a high positive correlation between control and treated samples. (b) Pie chart showing BLAST *e*-value distribution against NT database. (c) Pie chart showing species distribution as a percentage of total homologous sequences with an *e*-value <1 × 10^−5^ using the BLAST search against NT database. (d) Venn diagram showing common and uniquely expressed genes in control and treated samples.

We performed a comprehensive RNA-Seq profile of longan buds under the KClO_3_ treatment to explore the global gene expression changes in flower tissues. The transcriptome sequencing of the four buds RNA samples generated a total of 206.9 M clean reads of length 125 bp (paired-end), with an average of 172 M processed reads. An average of 75.56% of total reads passed ≥30 Phred score. The percentages of aligned reads were 76.85% and 74.26% for control and treated samples, respectively. The summary of reads and mapping statistics is presented in Table 1. The relation between gene expressions in all biological replicates indicates a high positive correlation between control and treated samples, respectively (Figure 2a).

**Table 1:** Summary of transcriptome analysis generated by RNA-Seq

Samples	fastq	Bases	Q20	Q30	GC	Average read length	Total number of clean reads	Percentage mapped reads to the reference genome
Control 1	R15050311_1 c1	1,917,358,250	95.35	90.82	44.08	125	17,090,142	77.52
	R15050311_2	1,917,358,250	94.22	89.23	43.99	125	17,090,142	76.95
Control 2	R15050315_1 c2	2,288,825,500	95.25	90.69	44.01	125	15,338,866	77.38
	R15050315_2	2,288,825,500	94.24	89.34	43.92	125	15,338,866	76.90
Control 3	R15050321_1 c3	2,036,296,250	95.24	90.68	44.74	125	18,240,475	76.44
	R15050321_2	2,036,296,250	94.2	89.24	44.65	125	18,240,475	75.93
Treated 1	R15050310_1 t1	2,136,267,750	95.37	90.99	44.2	125	18,310,604	76.12
	R15050310_2	2,136,267,750	93.94	89.03	44.09	125	18,310,604	75.68
Treated 2	R15050312_1 t2	2,280,059,375	95.54	91.16	43.88	125	18,215,170	74.30
	R15050312_2	2,280,059,375	94.15	89.15	43.79	125	18,215,170	73.81
Treated 3	R15050320_1 t3	2,276,896,250	95.37	90.9	43.83	125	16,290,370	73.06
	R15050320_2	2,276,896,250	94.1	89.11	43.74	125	16,290,370	72.61

A search performed against NCBI Nr database resulted in 21,493 genes with significant homology (*e-*value = 1 × 10^−5^). Analysis of *e*-value distribution for the sequence resulted from BLAST hit against NT database showed the even distribution ranging from *e*-value 0 to 10^−5^. More than 88.4% of sequences showed significant homology hits (*e*-value <1 × 10^−30^).

The species distribution result showed that 45.4% of best matching sequences was distributed among five plant species with *Theobroma cacao* (18.3%) and *Cephalotus follicularis* (7.6%), representing the top two species with the highest matching hits (Figure 2c).

Of 31,006 transcripts, a total of 21,493 genes were found to be expressed in control and treated samples with 701 and 2,143 unique genes in control and treated samples, respectively. A total of 18,649 genes were found common between both samples (Figure 2d).

### 3.2 Differential expression of genes

**Figure 3:**
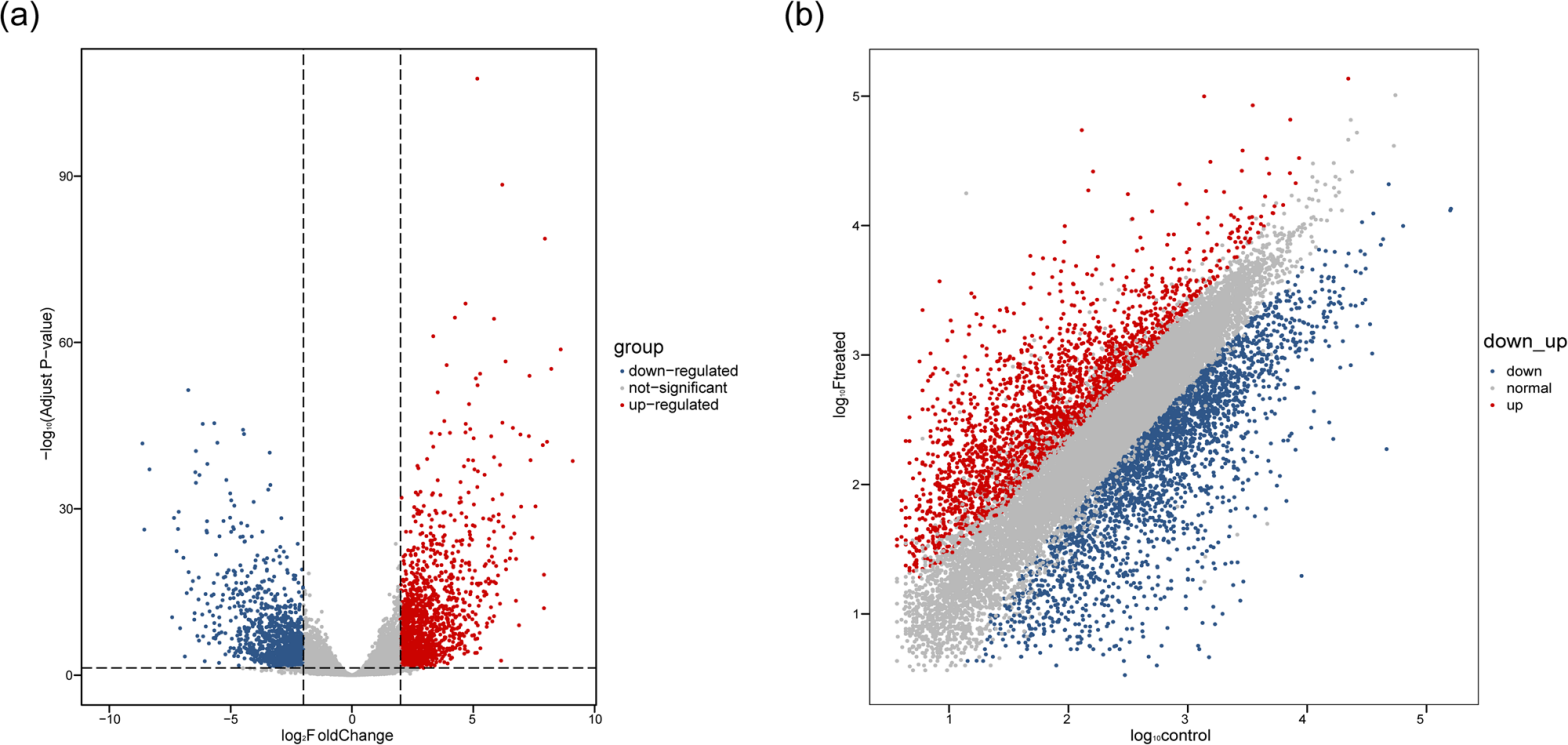
Distribution of upregualted and downregulated DEGs. (a) Volcano plot. (b) MA plot. The red and blue dots represent the upregulated and downregulated DEGs, respectively, in KClO_3_-induced floral buds as compared to untreated control (*p* adjust <0.05).

A total of 18,649 DEGs were identified between control and treated samples. The TPM values of several DEGs were observed to be <1; therefore genes with TPM ≥ 1 at least in one sample were also taken into consideration. Of 18,649 DEGs, only 4,904 genes were significant between control and treated groups, with 2,663 upregulated and 2,241 downregulated genes, indicating that the number of upregulated genes was higher in KClO_3_-induced flower buds. The total numbers of upregulated and downregulated DEGs (adjusted *p*-value ≤0.05) are represented in the volcano plot and MA plot (Figure 3a and b). To explore the potential functions of DEGs, all the transcripts were further annotated using UniProt databases.

### 3.3 GO and KEGG enrichment analysis

**Figure 4:**
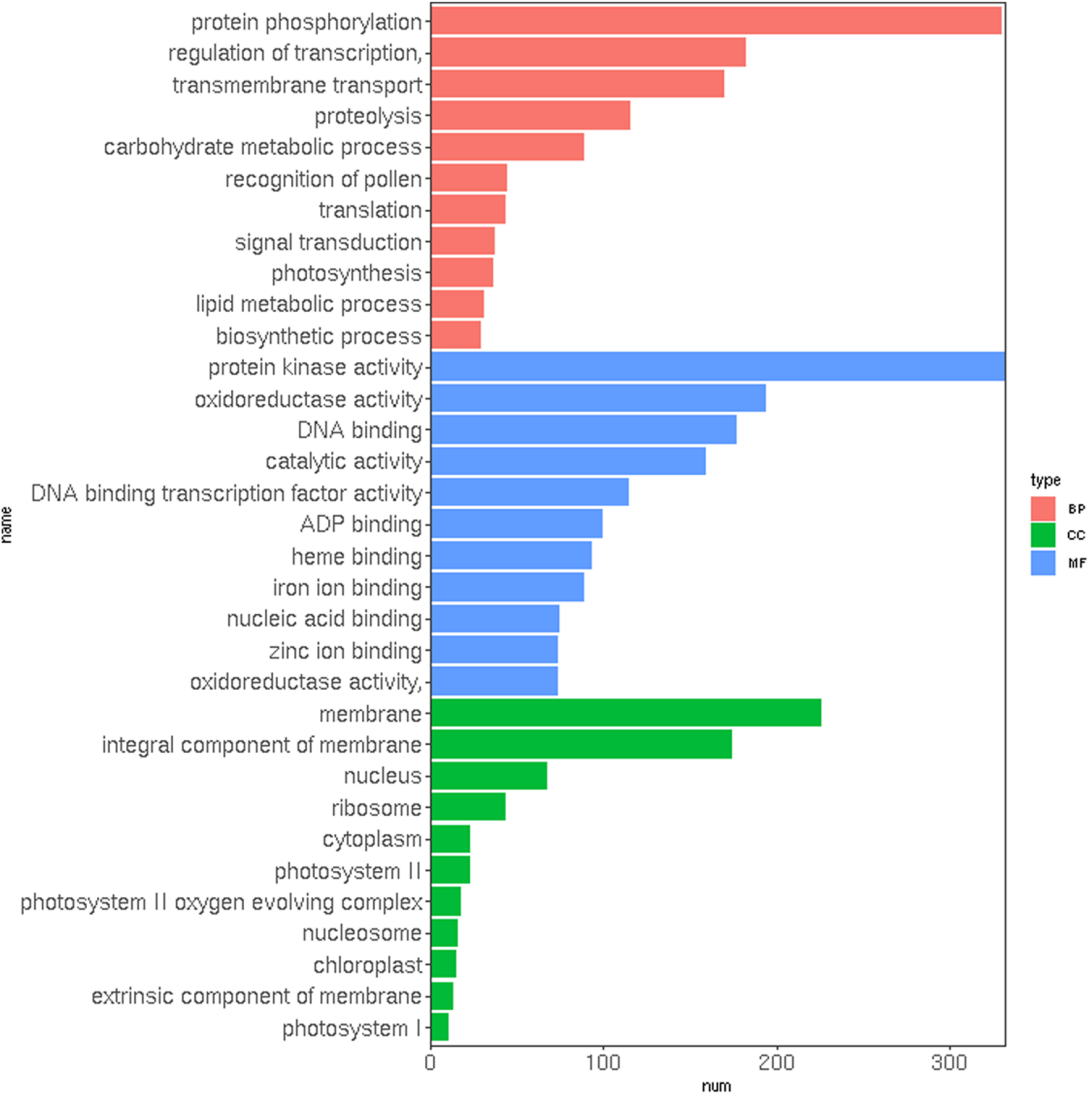
Gene ontology distribution of upregulated DEGs in KClO_3_-induced floral buds. The top 10 enriched GO terms are shown in the *y*-axis and categorized into the BP, MF, and CC groups. The *x*-axis indicates the number of DEGs.

The DEGs were classified according to GO terms of the BP, cellular component (CC), and molecular function (MF) and were distributed among 33 GO categories. In the BP category, the highest number of DEGs were involved in “protein phosphorylation” and “regulation of transcription.” While in the CC and MF categories, most DEGs were assigned to “membrane” and “protein kinase activity,” respectively (Figure 4). Among the upregulated significant GO term in the BP category, terms related to “regulation of transcription,” “diterpenoid biosynthetic process,” and “hydrogen peroxide catabolic process” were the most enriched. In the CC category, “nucleus” and “nucleosome” were overrepresented, whereas “DNA binding,” “ADP binding,” and “terpene synthase activity” were the most enriched terms in the MF category. On the other hand, “photosynthesis,” “photosystem II,” and “structural constituent of ribosome” were among the downregulated significant GO term in the BP, CC, and MF categories, respectively. The results of GO enrichment analysis to identify the major gene groups in longan flower buds are provided in File S3 and Figure S1.

**Figure 5:**
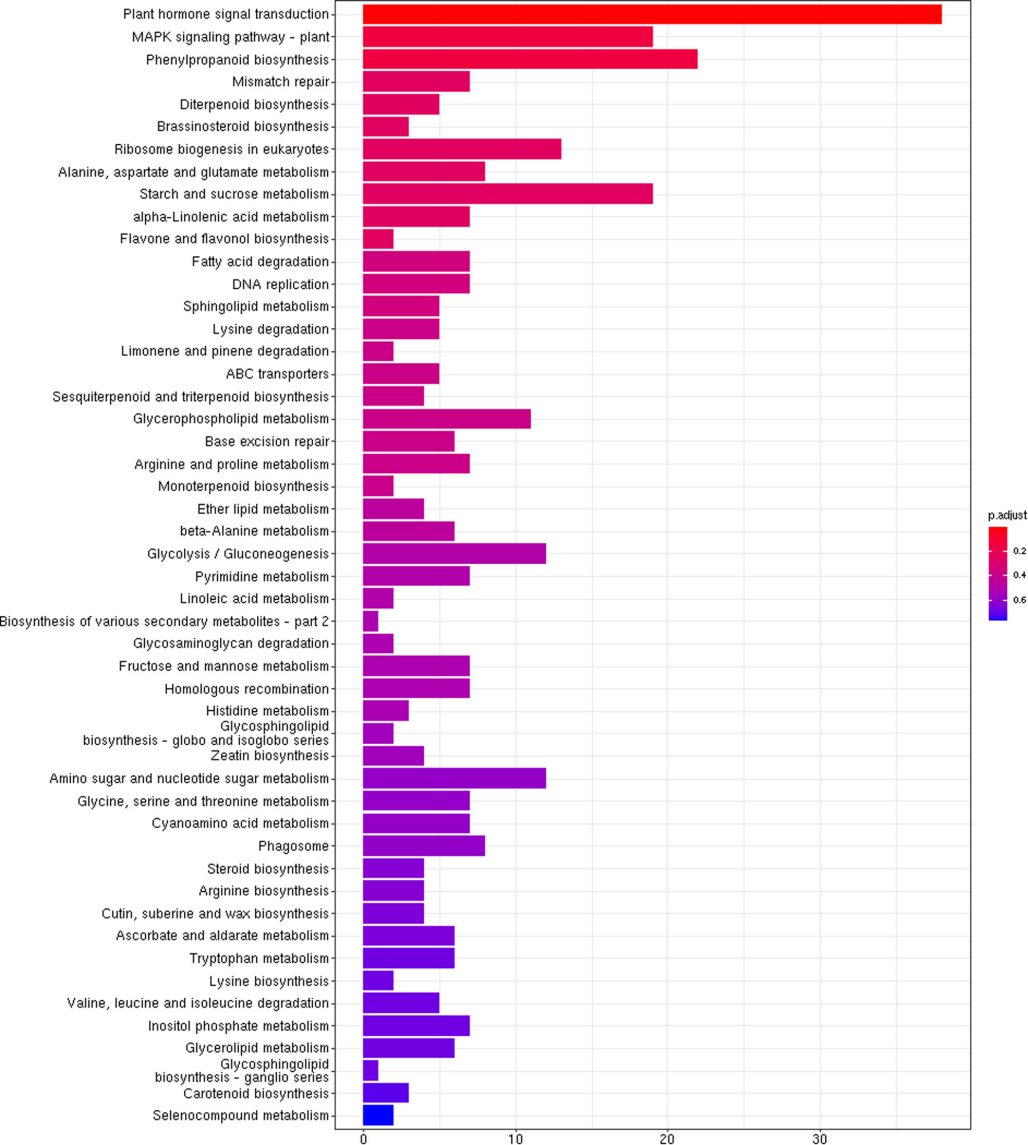
KEGG pathway assignments of upregulated DEGs in KClO_3_-induced floral buds. The *x*-axis indicates the number of DEGs. The *y*-axis represents the pathways (*p* adjust < 0.05).

Furthermore, the KEGG pathway analysis revealed the molecular interactions among the DEGs that are enriched in various metabolic pathways. In this analysis, DEGs were classified into 50 upregulated and 50 downregulated functional categories. In “plant hormone signal transduction” pathway, DEGs related to ethylene-insensitive protein 3/transcription factor *TCP21* (Dlo_030832.1), *SAUR* family protein|auxin responsive *GH3* gene family (Dlo_024340.1), phosphorelay signal transduction system (Dlo_012378.2), pto-interacting protein 1 (Dlo_014807.1), abscisic acid (ABA)-responsive element binding factor (Dlo_010957.1), and salicylic acid (SA)-mediated signaling pathway/E3 ubiquitin-protein ligase *SIAH1* (Dlo_000903.1) have been identified. Group “MAPK signaling pathway – plant” (e.g., chitinase (Dlo_024177.1), ATP-dependent RNA helicase *DHX36* (Dlo_017595.1), pto-interacting protein 1 (Dlo_014807.1), “phenylpropanoid biosynthesis” (e.g., tropinone reductase (Dlo_021546.1), 2-oxoglutarate dehydrogenase E2 component (Dlo_021361.2)), and “Mismatch repair” (e.g., DNA topoisomerase III (Dlo_000018.1), DNA ligase 1 (Dlo_030125.2)) were significantly enriched among the upregulated DEGs (Figure 5 and Figure S2). Apart from that DEGs were also enriched in the “starch and sucrose metabolism” pathway. On the other hand, “photosynthesis” (e.g., photosystem II oxygen-evolving complex (Dlo_000140.1), light-harvesting complex II chlorophyll a/b binding protein 7 (Dlo_034774.1)), “porphyrin and chlorophyll metabolism” (e.g., ubiquinol-cytochrome c reductase cytochrome b subunit (Dlo_0103421), NADH dehydrogenase (ubiquinone) flavoprotein 2 (Dlo_028554.1)), and “carbon fixation in photosynthetic organisms” pathways (e.g., fructose-bisphosphate aldolase activity (Dlo_024376.1), pyruvate dehydrogenase E1 component (Dlo_031702.1)) were the most enriched pathways among the downregulated DEGs (Figures S3 and S4). The enrichment analysis of KEGG pathways is provided in File S4.

### 3.4 Identification of DEGs related to flower induction and development

**Figure 6:**
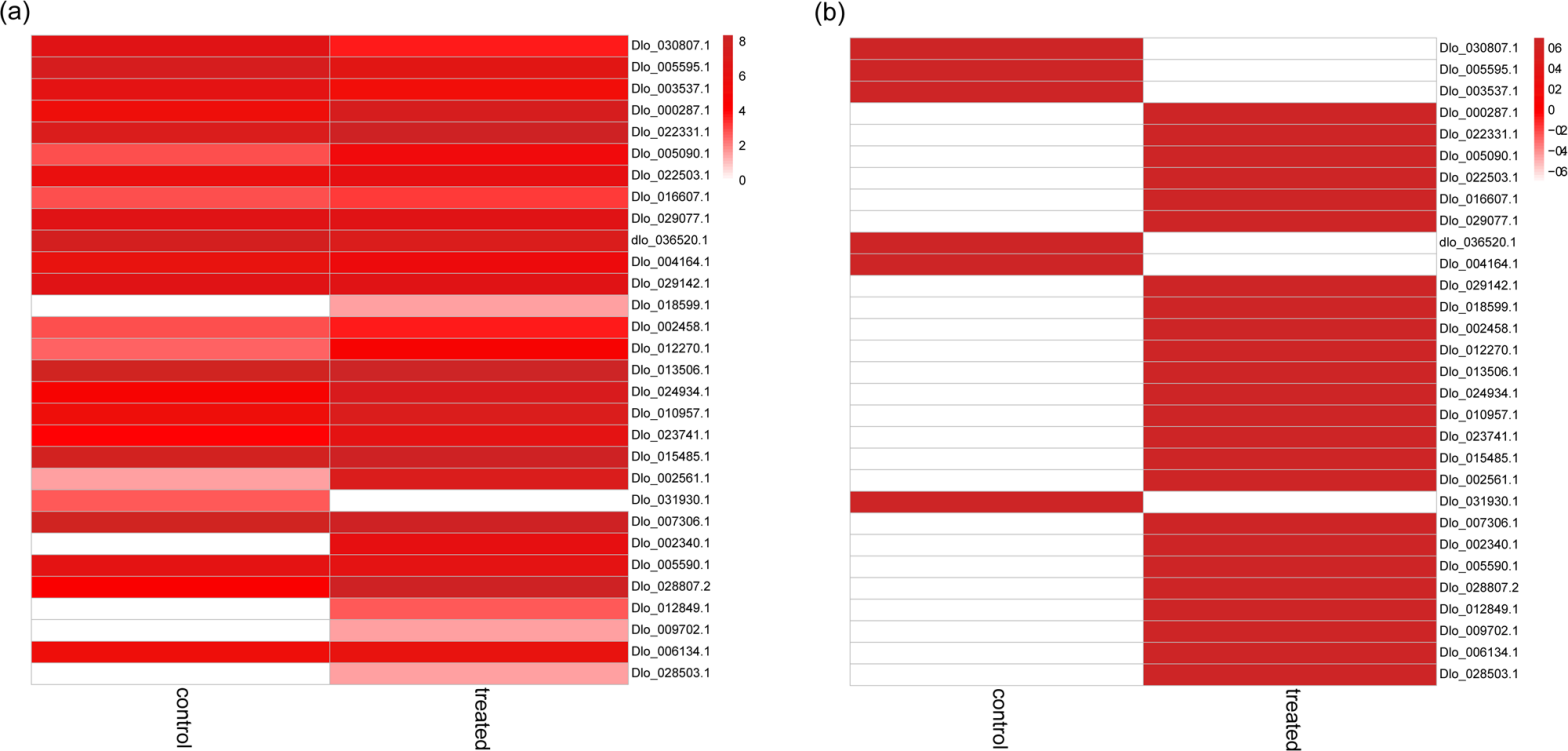
Heatmap representing the differential expression of flowering-related genes between untreated control and KClO_3_-induced floral buds. (a) Heatmap of non-normalized expression values. (b) Heatmap of row-normalized expression values.

A heatmap representing the 29 flowering-related DEGs was generated to analyze the comparative expression of genes between control and treated flower samples (Figure 6a, b). Among these DEGs, 13 genes were upregulated in response to KClO_3_ treatment. DEGs related to *AP2* Dlo_000287.1 (Log2FC 2.745), *ARF3/ETTIN* Dlo_022331.1 (Log2FC 1.166), *BLADE ON PETIOLE 1* (*BOP1*) (Dlo_005090.1; Log2FC 3.045), *PENNYWISE* (*PNY*) (Dlo_024934.1; Log2FC 3.799), *PERIANTHIA* (*PAN*) (Dlo_010957.1; Log2FC 2.170), PETAL *LOSS* (*PTL*) (Dlo_023741.1; Log2FC 3.003), and *SPL8* (Dlo_028807.2; Log2FC 5.18) were shown significant uplregulation (Figure 6b). However, DEGs related to *AG* (Dlo_030807.1; Log2FC −4.222), *AGL42* (Dlo_005595.1; Log2FC −1.615), *AP1* (Dlo_003537.1; Log2FC −1.718), and *HISTONE DEACETYLASE 1* (*HD1*) (Dlo_036520.1; Log2FC −0.879) were significantly downregulated. Moreover, DEGs correspond to *LATE MERISTEM IDENTITY 1* (*LM1*) (Dlo_002458.1), *BLADE ON PETIOLE 2* (*BOP2*; Dlo_022503.1). *LEUNIG* (*LUG*; Dlo_013506.1), *SEUSS* (*SEU*; Dlo_007306.1), and *ULTRAPETALA1* (*UTL1*; Dlo_006134.1) were also found to be induced in our data.

**Figure 7:**
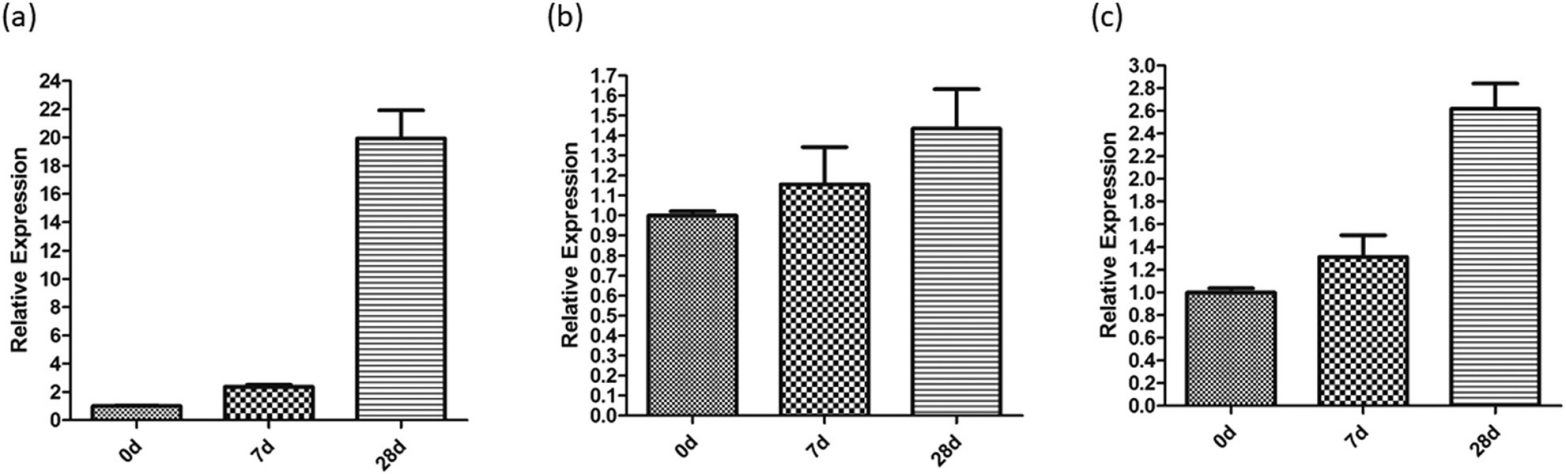
Expression levels of the longan genes at 0, 1, and 4 weeks after the treatment measured by quantitative RT-PCR. (a) Expression levels of *AP2*; (b) *ARF3/ETTIN* expression levels; and (c) *BOP1* expression level.

To confirm the gene expression results based on transcriptomic quantification, we measured the expression levels of *AP2* (Figure 7a), *ARF3/ETTIN* (Figure 7b), and *BOP1* (Figure 7c) using qRT-PCR. We sampled buds with the treatment of KClO_3_ at 0, 1, and 4 weeks, respectively. All the genes demonstrated elevated expression, agreeing with the transcriptomic results. Expression of all the genes showed higher expression levels at 1 week after the treatment than the starting point, and at 4 weeks than 1 week, suggesting continuous elevation of the gene expression with the treatment. The *AP2* (Figure 7a) expression showed the elevation of 20-folds at 4 weeks after the treatment, which was particularly striking.

## 4 Discussion

The floral induction is crucial for the production of subtropical fruit trees such as longan, which depends on the temperature, nutrient availability, etc. To optimize the off-season flowering in longan trees, many cultural management techniques have been employed. The applications of chemicals such as KClO_3_, sodium chlorite, and sodium hypochlorite have demonstrated the off-season floral induction in longan [9]. In the present study, we performed a comparative transcriptome of vegetative and flower buds induced in response to KClO_3_ treatment to identify the differentially expressed genes in longan. A total of 4,904 DEGs out of 18,649 genes were identified as significant in our dataset, and among them, 2,663 were upregulated and 2,241 were downregulated genes.

FM establishment and maintenance, organ initiation, flower transition, and morphogenesis depend on the tight regulation of a complex gene regulatory network. This complex network involves the sequential and coordinated function of TFs, which control the floral transition and development [10]. The GO analysis revealed that most DEGs were involved in the regulation of transcription, nucleus, DNA binding, and ADP binding. The highly induced DEGs such as *Apyrase* (Dlo_027533.2), transcription factor *TCP21* (Dlo_032272.1), and *DNA topoisomerase III* (Dlo_009019.1) suggest that these genes actively participate in the transcriptional regulation of flowering-related genes. These results are consistent with the results of recent publications [11,12,13,14], suggesting similar mechanisms in different plant species.

Plant hormones play a key role in the regulation of plant growth and development. In KEGG pathway analysis, several DEGs involved in “plant hormone signal transduction” such as *EIN3*/transcription factor *TCP21*, *SAUR* family protein/auxin-responsive *GH3* gene family, phosphorelay signal transduction system, pto-interacting protein 1, *ABF*, and SA-mediated signaling pathway/E3 ubiquitin-protein ligase *SIAH1* have been identified. Ethylene levels in the plant can influence the regulatory network that controls the flowering timing [15]. EIN3 acts as an activator of ethylene-inducible genes. The regulatory role of *ein3-1* mutants was observed in the transition from vegetative to reproductive growth in *Arabidopsis* [16]. The expression of auxin-responsive *GH3* gene family and *SAUR* family protein was observed to be differentially regulated during floral transition in two longan cultivars and speculated to be involved in perpetual flowering by regulating the FM [17]. ABA also plays an important role in floral transition as previously reported [18]. In our study, a gene related to *ABF* was upregulated, indicating that the ABA signaling pathway might regulate the floral induction in longan in response to the KClO_3_ treatment. Similarly, the SA signaling pathway also affects flower induction [19]. A gene involved in SA-mediated signaling pathway (Dlo_000903.1) was also induced in our study. The aforementioned result suggests that these genes might play a critical role in the flowering-related signaling pathway via hormone regulation in the longan bud initiation process.

Further, mitogen-activated protein kinase (MAPK) pathways play a crucial role in environmental and developmental signal transduction. MAPKs are involved in various cellular processes and regulate plant hormone signaling pathways via molecular crosstalk. Ethylene signaling is influenced by MAPK-dependent phosphorylation and activation of transcription factor EIN3 [20]. Several genes related to the MAPK signaling pathway were induced in the present study such as *phosphatidylinositol phospholipase C* (Dlo_016250.2), *phosphoglycerate kinase* (Dlo_001972.1), *5-amino-6-(5-phospho-d-ribitylamino) uracil phosphatase* (Dlo_021916.1), *S-phase kinase-associated protein 1* (Dlo_011083.1), phosphorelay signal transduction system (Dlo_012378.2), and *pto-interacting protein 1* (Dlo_014807.1), indicating that MAPK signaling controls the flowering via crosstalk between plant hormone signaling and flowering-related pathway in longan. MAPK has been shown to be related to other physiological processes in flower, such as heat-stress response [21] and self-incompatibility [22], but the rare report relates MAPK with chemical treatment-induced expression MAPK. This indicates that MAPK might be involved in other flowering-related physiological processes.

Carbohydrates are an important source of energy for plant development and other cellular processes. The previous study has shown a higher rate of sucrose export during floral induction in *Arabidopsis* [23]. In a study of comparative expression analysis of two longan varieties, genes related to *trehalose-6-phosphate synthase* (*TPS1*), *granule bound starch synthase 1* (*GBSS1*), and *starch synthase 2* (*SS2*) have shown downregulation in longan genotype with PF trait suggested that these genes act as an inhibitor of PF traits in longan [17]. TPS1 is required for flower induction in *Arabidopsis* and apple, and the loss of TPS1 function causes a delay in flowering [24]. We observed several DEGs related to starch and sucrose metabolism and carbohydrate metabolism in this study such as *glucan endo-1,3-beta-glucosidase* (Dlo_024378.1), trehalose biosynthetic process (Dlo_012338.1), *gluconokinase* (Dlo_001453.1), *beta-fructofuranosidase* (Dlo_013696.1), *UDP-glucose--hexose-1-phosphate uridylyltransferase* (Dlo_035054.1), and *mannose-1-phosphate guanylyltransferase* (Dlo_022083.1), which indicate the importance of carbohydrate metabolism regulation during the flower development in longan. Our results also identified the DEGs involved in “phenylpropanoid biosynthesis” and “diterpenoid biosynthesis” pathways such as *tropinone reductase* and *thujopsene synthase*, which were highly induced. It was reported that floral terpene volatiles play an important role in the plant development [25].

Environmental signals including photoperiod, temperature, age, and nutrient supply can affect the floral transition involving the conversion of shoot apical meristem (SAM) into an inflorescence meristemleading to flower initiation [18]. These pathways trigger the floral integrator genes such as *FT* and *SOC1*, which in turn activate the FM identity genes *LFY* and *AP1*. In our study, the expression of *LFY*, *FT*, and *SOC1* was not detected as observed by Jia et al. [26]. However, in contrast to the previous study [27], *AP1* expression showed downregulation in our result. On the other hand, we observed upregulation in gene-related to *AP2*, which is a member of the AP2/ethylene response factor (ERF) transcription factor family and a target of miR172 involved in floral stem cell control [28]. It is reported that the AP2 restricts the expression of *AG* during the regulation of floral stem cells in *Arabidopsis*. Previous reports demonstrated that *AP2* expression was downregulated in longan [17,26]; however, AP2 seems to control the regulation of flowering genes in KClO_3_-induced floral buds in our study. Further, genes related to *AG* and *AGL42* were downregulated supporting the previous finding. An SBP box domain TF-related gene *SPL8* was highly upregulated in this study. *SPL8* and other miR156-targeted *SPL* genes play a crucial role in anther development and gynoecium patterning [29]. Both AP2 and SPL are involved in the regulation of age-dependent response to vernalization [30]. ARF3/ETTIN is involved in the regulation of expression of auxin-responsive genes as well as cytokinin biosynthesis genes and plays a role in leaf polarity specification and floral organ patterning [31]. It was observed that ARF3 participates in FM determinacy by repression of *WUSCHEL* (*WUS*) gene expression, a central player in FM determinacy. AP2 and AG control the action of WUS by the activation and repression of *WUS* expression, respectively. In *Arabidopsis*, ARF3 acts as a target of AP2 and integrates the function of AP2 and AG in FM determinacy [32]. Interestingly, *ARF3* expression is upregulated in our study, which indicates that ARF3 peculiarly coordinates with AP2 and AG to regulate the KClO_3_-induced flowering in longan. It was reported that another player that regulates the floral stem cell termination is UTL1, which induces the *AG* expression and MADS-Box genes during flower development. It was revealed that UTL1 genetically interacts with LFY via the regulation of *AG* and MADS box genes; however, UTL1 and LFY appear to act independently to regulate genes involved in floral organogenesis [33].

A gene related to *BLR* or *PNY*, which belongs to a BELL homeodomain transcription factors family, was shown a higher fold of induction. A study showed that BLR/PNY has a role in the maintenance of SAM and FM specification as well as the suppression of *AG* [34]. Similarly, a bZIP transcription factor, PAN, binds and activates the expression of *AG* in the center of FM [35]. We observed the upregulation in *PAN* expression in our data. A gene related to *LMI1* was also found to be upregulated. LMI1 is activated by LFY and activates the *CAULIFLOWER* (*CAL*), another FMI gene, which ultimately induces *AP1* expression together with LFY [36]. Grandi et al. [37] observed that AP1 represses the *LM1* expression, indicating that a regulatory feedback loop is required to maintain the relative expression of these genes to establish the FM identity. However, little is known about whether the BELL transcription factor is involved in flowering initiation, although our results suggest its role in flowering initiation in longan. This indicates its value for further studies to confirm its role in such processes.

Next, LUG and SEU, two transcriptional co-repressors interact with AP1 and SEP3 and repress the *AG* expression in outer floral whorls [38]. Both *LUG* and *SEU* showed upregulation in this study. Transcriptional co-regulators, BOP1 and BOP2, regulate the architecture of leaves, fruits, and flowers. Loss-of-function mutant *bop1 bop2* revealed defective inflorescence and floral architecture [39]. A study showed that BOP1/2 interacts with PAN and induces the expression of *AP1* in floral primordial as well as downregulates *AGL24*. BOP1/2 activates *AP1* in FM in an LFY-independent manner [40]. We observed upregulation in *BOP1/2* expression in our study. PTL is another regulator in flower development, which showed higher expression in this study and was reported to be expressed in boundaries between sepal primordial and restricts growth between newly formed sepals [41].

Histone modification plays an important role in flower development. In *Arabidopsis*, an antisense *AtHD1* showed ectopic expression of tissue-specific gene *SUPERMAN* (*SUP*) in floral whorls with aberrant phenotype and other flower defects [42]. In another study, it was observed that *HUA ENHANCER 3* (*HEN3*) encoding cyclin-dependent kinase E plays a role in the specification of stamen and carpel identity, whereas *HEN4* plays a role in delaying flowering by activating repressor genes *FLC* and *MADS AFFECTING FLOWERING 4* (*MAF4*) [43]. In our study, genes related to *HD1*, *HEN3*, and *HEN4* have shown downregulation, suggesting that these genes regulate the expression of flowering-related genes in KClO_3_-induced longan flower differently as compared to *Arabidopsis*.

The expression of floral integrators *FT*, *SOC1*, and *LFY* is absent in our results, whereas *AP1* expression is downregulated. Moreover, the expression of the key regulators in floral transition, *FLC*, and *TFL1* could not be detected in this study. The absence of *FT* expression in longan bud is consistent with the previous study, where *FT* expression was only detected in mature leaves during floral induction. We speculate that the floral induction pathway in KClO_3_-induced longan floral bud is primarily associated with the activation of AP2 and suppression of AG. The factors such as ARF3, BLR/PNY, LUG, SUS, and PAN regulate the expression of *AP2* and *AG* for FM specification and determinacy, whereas genes related to *LMI1*, *BOP1*, *BOP2*, and *UTL1* might control the expression of *AP1* and *AG* via influencing *LFY* expression through a regulatory feedback loop.

In conclusion, our comparative transcriptome analysis of KClO_3_-induced flowering in longan revealed that the synergistic action of flowering-related genes and genes involved in plant hormone signaling, MAPK signaling cascades, and carbohydrate metabolism is required for FM determinacy in longan buds. Further studies involving specific development stages of floral buds in a temporal-spatial manner are required for future work, which might explain the exact role of these genes in flower induction in off-season longan. Our study provides a detailed insight into the molecular mechanisms underlying KClO_3_-induced floral induction in longan which will offer a platform to study flower development for improved production in off-season fruit trees.

## Supplementary Material

Supplementary Figure
