# Supplementary material for: Chlorate-induced molecular floral transition revealed by transcriptomes
Source: Open Life Sci. 2023 Jul 29;18(1):20220612. doi: 10.1515/biol-2022-0612 (PMC10389677; doi:10.1515/biol-2022-0612)
Supplement: Supplementary Figure [file biol-2022-0612-sm.pdf]

Supplementary material

File S1: The annotation results of all DEGs (xlsx).
File S2: List of flowering-related DEGs (xlsx).
File S3: GO term analysis of DEGs (xlsx).
File S4: KEGG pathway enrichment analysis of DEGs (xlsx).

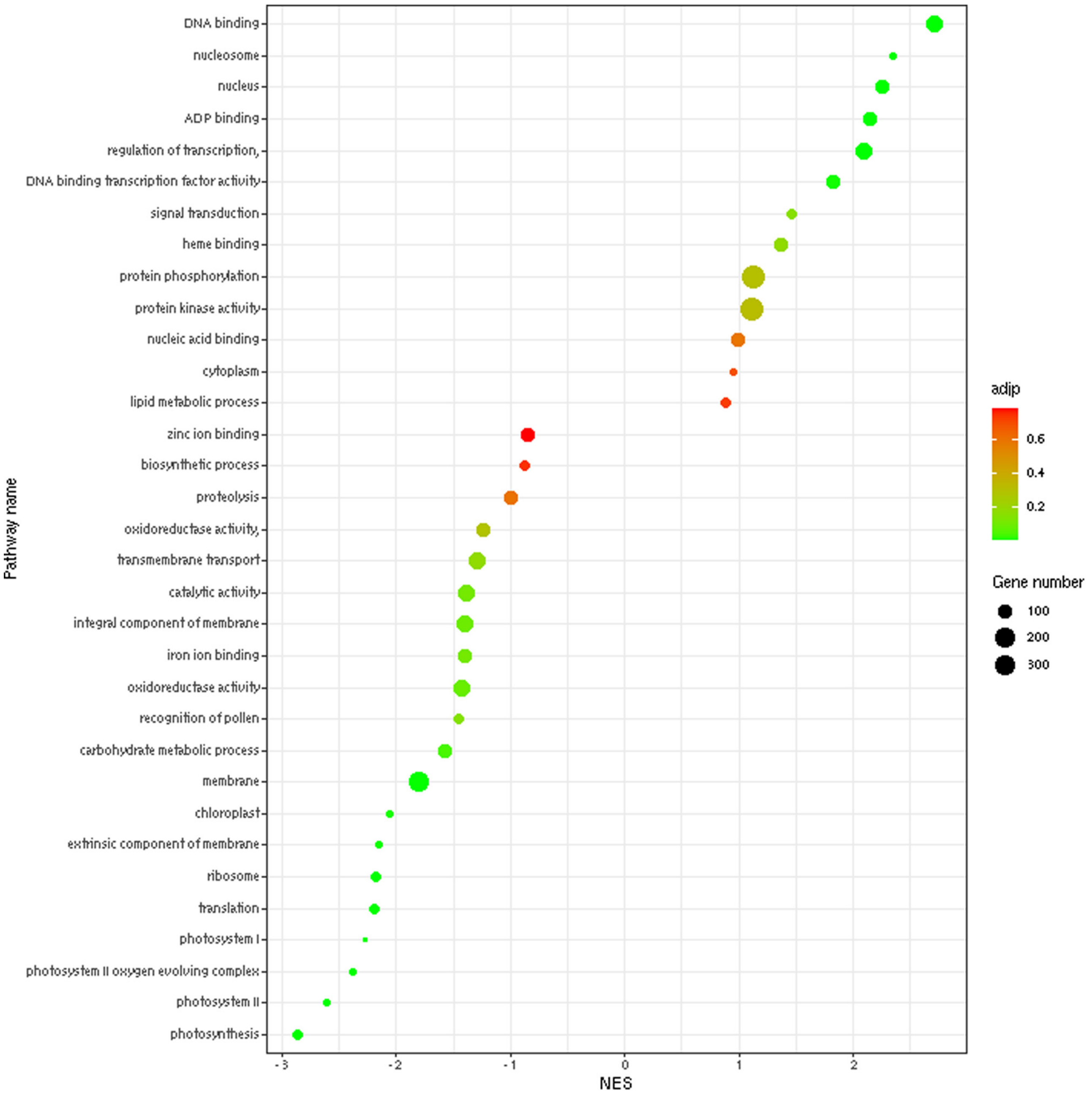

Figure S1: Dot plot representation of significantly enriched GO terms.

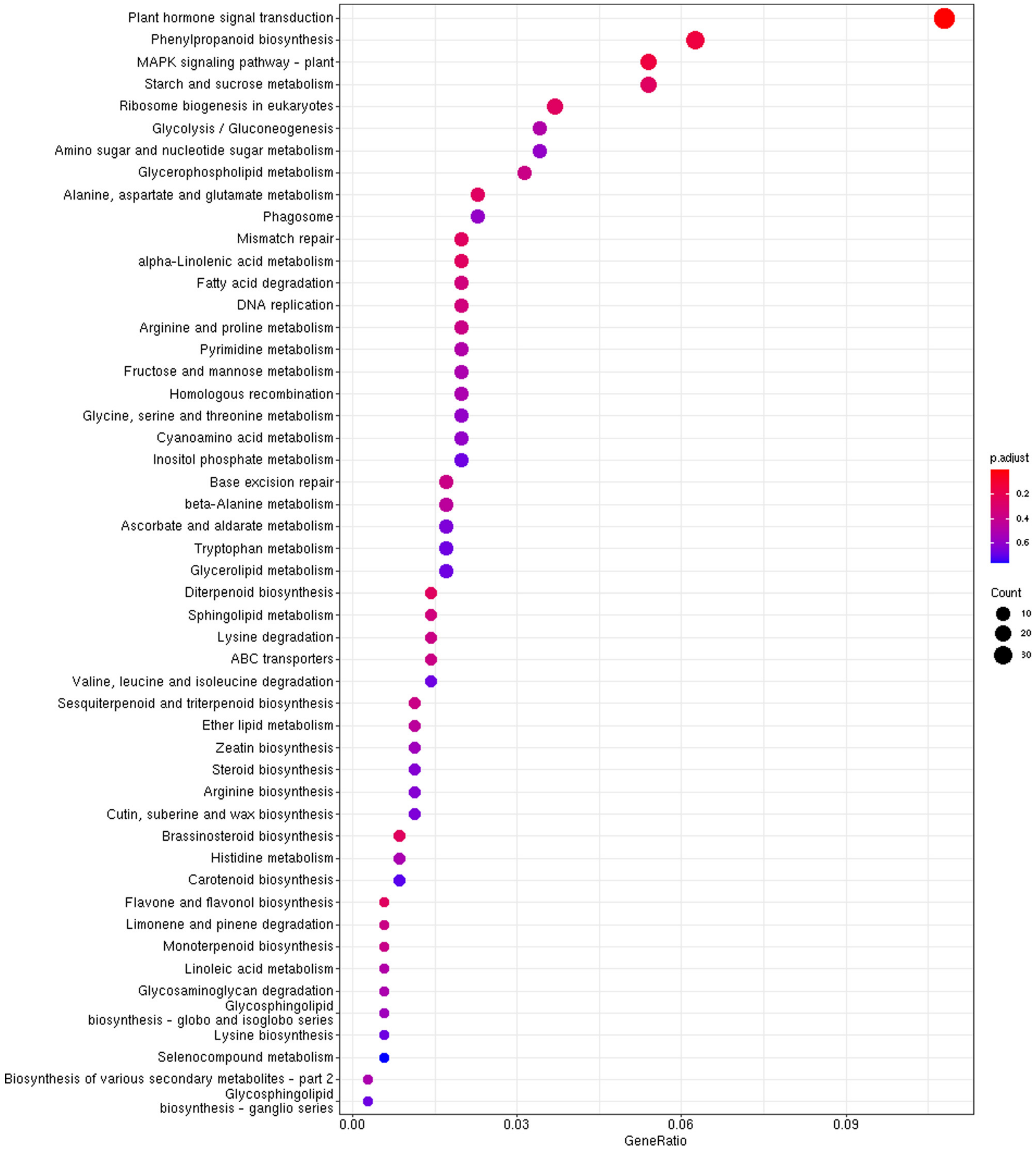

Figure S2: Dot plot representation of significantly enriched KEGG pathways of upregulated DEGs.

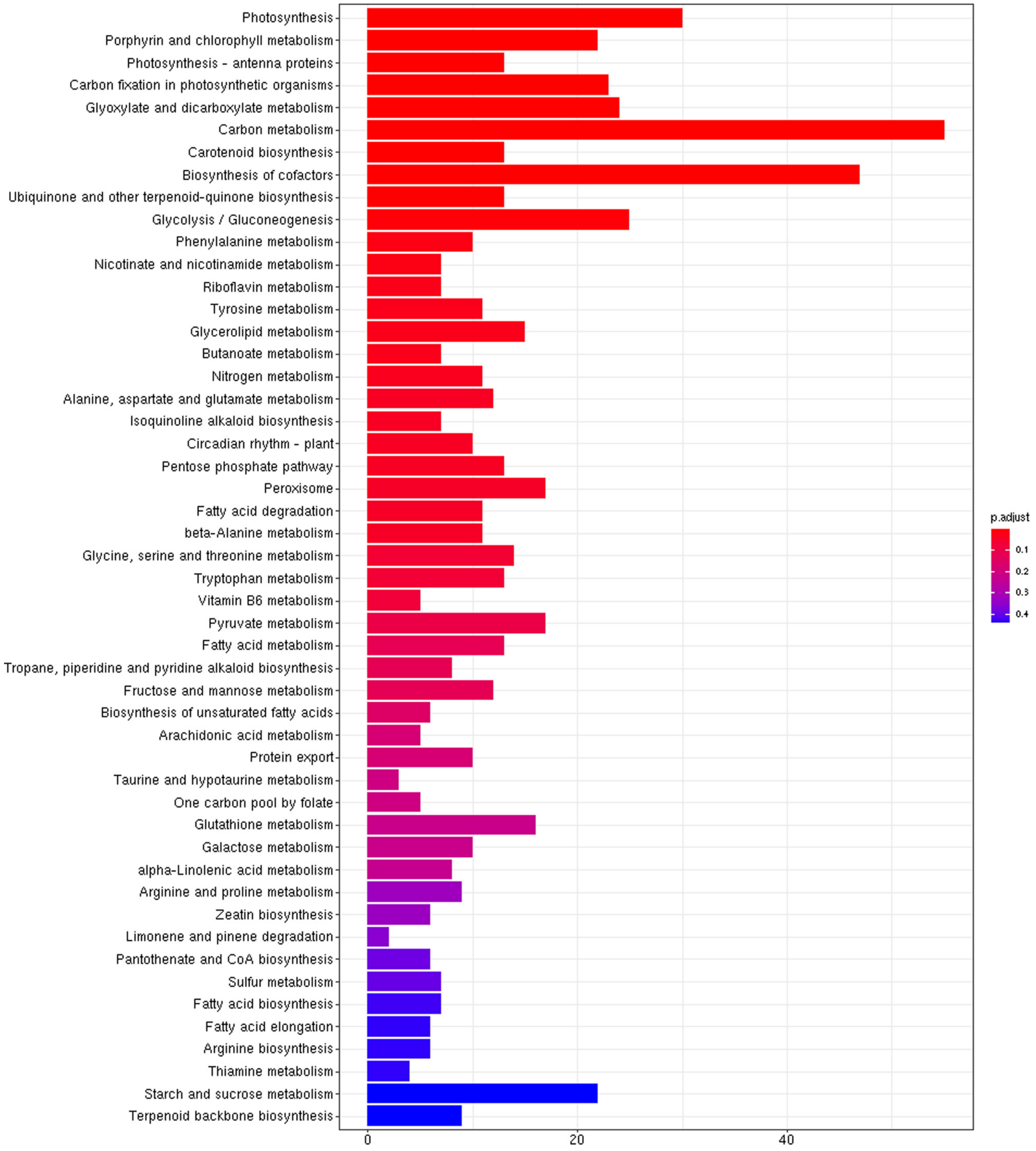

Figure S3: Bar plot representation of significantly enriched KEGG pathways of downregulated DEGs.

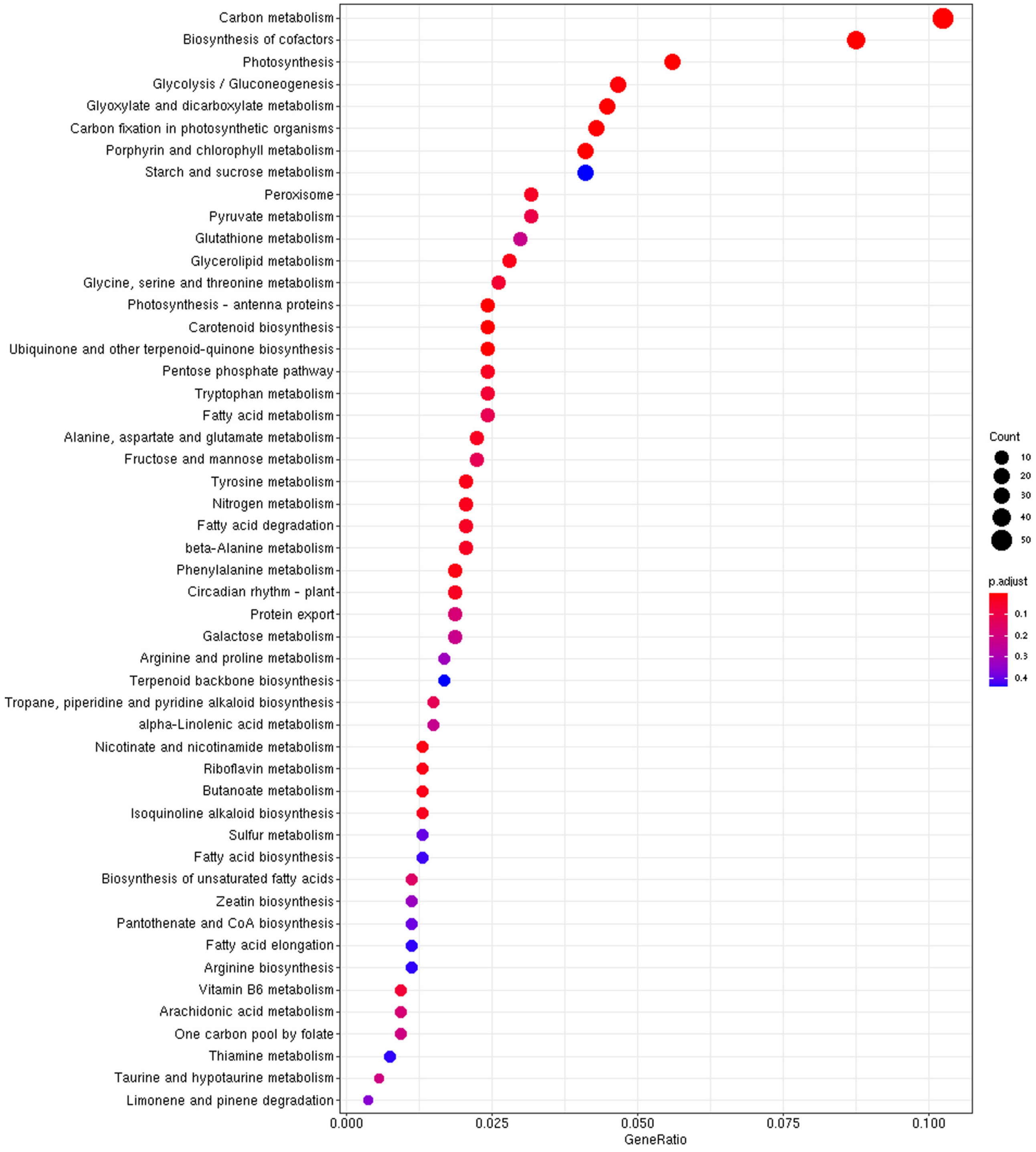

Figure S4: Dot plot representation of significantly enriched KEGG pathways of downregulated DEGs.
